# The microbiological and sensory status of dual-purpose chickens (Lohmann Dual), male Lohmann Brown Plus chickens, and conventional laying hens slaughtered in a laying hen abattoir compared to conventional broilers slaughtered in a broiler abattoir

**DOI:** 10.1371/journal.pone.0277609

**Published:** 2022-11-14

**Authors:** Nina Langkabel, Verena Oswaldi, Roswitha Merle, Cornelia Fleischhauer, Cathleen Heinke, Thomas Alter, Lüppo Ellerboek, Reinhard Fries, Diana Meemken

**Affiliations:** 1 Institute of Food Safety and Food Hygiene, Freie Universität Berlin, Berlin, Germany; 2 Institute of Veterinary Epidemiology and Biostatistics, Freie Universität Berlin, Berlin, Germany; 3 German Agricultural Society (DLG Test Service GmbH), Frankfurt am Main, Germany; 4 Federal Ministry of Food and Agriculture, Berlin, Germany; Tokat Gaziosmanpasa Universitesi, TURKEY

## Abstract

Alternatives to conventional chicken meat and egg production are increasingly under discussion, especially because of the common practice of killing male day-old chicks from laying lines which has been banned from the beginning of 2022 in Germany and is planned to be banned during 2022 in other countries. Production of dual-purpose chicken lines is one possible solution, as such lines combine moderate laying and growth performance. The microbiological status of products from such breeds must be comparable to existing products on the market for food safety purposes. Additionally, the production of such products will take longer because of the feeding regimes required, and again, comparability should be safeguarded for the best consumer protection. The dual-purpose chicken line, Lohmann Dual (males), was compared to males from the laying line Lohmann Brown Plus, conventional laying hens (all slaughtered and processed in the same conventional laying hen abattoir), and conventional broilers (slaughtered in a conventional broiler abattoir). Neck skin samples were taken before chilling at the end of each slaughter line to determine the microbial status of the carcasses. Additionally, fresh and cooked meat sensory analysis was performed on meat from broilers and male and female Lohmann Dual and Lohmann Brown Plus chickens (for three carcasses of each group) at the German Agricultural Society Test Center in Kassel. The focus was on the performance of male Lohmann Dual compared to the other lines. There was no difference in the *Enterobacteriaceae* count of the dual-purpose chicken line compared to conventional broilers, whereas laying hens had a significantly higher microbial load before chilling, as based on neck skin examinations (p<0.001). According to sensory test results, the meat from dual-purpose chickens was the best (as no defects were found) among the five chicken meat types examined. In conclusion, based on their microbial status and sensory analysis of fresh and cooked meat, Lohmann Dual males slaughtered in a laying hen abattoir can be considered as an alternative to conventionally kept broilers slaughtered in a broiler abattoir.

## Introduction

Negative genetic correlations between growth and reproductive traits are known in different livestock species [1]. In poultry, this fact has led to separated meat-type and egg-type lines [2, 3]. While chickens were used for dual-purpose until the 1920s, the intensive hybrid breeding of specialized meat and egg lines became standard for industrialized production since the Second World War [4, 5]. Over the last 50 years, breeding resulted either in special broiler lines with massive breast muscles as the main product or in specialized laying hens with high egg production [3, 6]. As the raising and fattening of male layer birds with only limited genetic potential for the purpose of growing [7] is mostly considered as economically not feasible, this development caused the killing of one-day-old male chicks from layer lines [6, 8–11]. In Germany, around 45 million male one-day-old chicks from laying lines are killed each year [12]. Due to ethical and animal welfare concerns, this practice is no longer accepted by the public and in general. Therefore, discussions and research are being conducted to find alternatives [13–20]. In Germany, this has ultimately led to a legal ban on the killing of male one-day-old chicks starting in January 2022 [21, 22]. In France, this practice is also planned to be banned by the end of 2022 [23]. In other countries, such as Switzerland (personal communication BVL Schweiz 2022) or the USA [24], there are also efforts to abolish this practice, although this is currently still being pursued at the level of the layer farming associations. More countries, like Austria, Ireland, Luxembourg [25], and Portugal (personal communication Susana Pais de Carvalho dos Santos 2022), are in favor of a ban on killing, but are still waiting for scientific evidence on alternative methods in order to reduce the number of male chicks being killed. Alternatives include in-ovo sexing, the use of dual-purpose chicken lines for egg and meat production, and the rearing of male chicks from laying lines [11, 26].

Process controls in poultry abattoirs are performed additionally to hazard analysis and critical control point (HACCP) programmes; these plans both contribute to poultry meat hygiene and safety. In this framework, neck skin samples are taken to assess the microbial status of the carcasses because microbial loads on neck skin after processing correlate with microbial loads on carcass surfaces, and thus, are used to represent the loads on surfaces of carcasses hanging head down [27]. Additionally, it is a very practical way of sampling without taking carcasses out of the slaughter line [28]. Moreover, for sampling poultry carcasses, skin tissue removal was shown to be the most precise and accurate sampling method to represent the skin contamination of poultry carcasses rather than the cotton swabs that were used by Avens and Miller [29]. Furthermore, neck skin samples are the standard sample types taken to assess process hygiene criteria (i.e., *Salmonella* and *Campylobacter*) in broiler abattoirs in the EU (Reg. (EC) No. 2073/2005 [30]).

The sampling location at the end of the slaughter line prior to chilling should be chosen if comparison between abattoirs with different chilling methods is to be performed. Results of microbial analyses after immersion chilling are not comparable to studies where chilling is performed by air or spray chilling because there can be a certain washing effect, but also, cross-contamination in the chilling tank is possible. Some studies found immersion chilling, compared to other chilling methods, produced lower microbial loads on carcasses [31–34], but one study [35] found the opposite.

The present study was conducted in the framework of research on possible alternatives to the killing of one-day-old chicks from laying lines in the joint project “Integhof”. The aim of this joint project was to investigate the use of the dual-purpose line Lohmann Dual (LD) in comparison to laying hens, males from the layer line Lohmann Brown Plus (LB), and conventional broilers.

The main aim of our study was to determine the microbial status of neck skin samples, representing the carcasses at the end of the slaughter line, from dual-purpose chickens LD compared to male LB birds (a typical layer line), conventional laying hens (all three were slaughtered in the same abattoir for laying hens), and conventional broilers (slaughtered in a broiler abattoir). In addition, sensory testing was performed on three chicken carcasses per group (male and female LD, male and female LB, broilers) at a high-level sensory testing facility to determine any differences between fresh and cooked meat from the dual-purpose chickens and those meats from other lines.

## Materials and methods

### Animals included for microbiological examinations and slaughter line

Birds included in the microbiological investigation were male LD and male LB chickens and as control groups, conventionally kept laying hens and conventional broilers. The male LD, male LB and conventionally kept laying hens were slaughtered in the same laying hen abattoir. The broilers were slaughtered in a broiler abattoir.

From 2015 to 2017, in total 5,344 male LD and 6,589 male LB were fattened according to the requirements of the national regulation regarding animal welfare and husbandry of livestock [36] in three trials under standardized floor housing conditions enriched with perches and straw bales at the conventional holding of the Farm for Education and Research in Ruthe of the University of Veterinary Medicine Hannover, Foundation, Germany, as described by Siekmann et al. [37]. An animal testing application was not necessary for our study because sampling and examinations took place after the animals were slaughtered by conventional means following Reg. (EC) No. 1099/2009 [38] in a conventional laying hen abattoir (see details below).

A live weight of around 1,500 g was defined as the target slaughter weight for both fattening groups (LD and LB). Both groups were slaughtered on the same day, so the fattening group that reached this target weight first set the date of slaughter for both groups. As the male LD had better weight gains, they reached the target weight first and had a higher average slaughter weight compared to the male LB. After the fattening period, the male LD and male LB birds were loaded into conventional transport crates on a truck for transport and were slaughtered in a commercial abattoir for laying hens. This abattoir was chosen because pre-tests had shown that the carcasses did not fit in the machines of a fully automated broiler abattoir, and the evisceration processes would have been compromised had we used the broiler abattoir.

At the laying hen abattoir, the animals were stunned with an electrical water bath. At the boiler abattoir stunning was performed by carbone dioxide. At both abattoirs the animals were bled by neck incision, then carcasses passed through the scalding tank and were plucked. Afterwards, heads and feet were removed, and the carcasses were hung on the evisceration line for the steps of opening the body cavity, evisceration, and removal of other organs. Carcasses in both abattoirs then passed through inside-outside-washers. After that, in the broiler abattoir, the carcasses were spray chilled, but in the laying hen abattoir, the carcasses were released from the slaughter shackles and dropped into the immersion chiller.

Because of the described possible different effects observed for immersion chilling and the supposed washing effect, in preparation for the current study, we decided to sample at the end of the slaughter line after the last inside-outside-washer, but before chilling. Additionally, we needed to compare our findings in a laying hen abattoir with an immersion chiller to our findings in a broiler abattoir with a spay-chilling system, so clearly the only possible sampling position was immediately before the different chilling processes in the two abattoirs.

During processing, post-mortem findings were recorded by official veterinarians and auxiliaries following the EU legislation.

### Sampling and microbiological tests

A total of 75 neck skin samples each from male LD and male LB and 200 neck skin samples from laying hens as control were taken directly after a final inside-outside-washer before the carcasses were dropped off the shackles into the immersion chiller in the laying hen abattoir. In the broiler abattoir, in total, 300 neck skin samples were taken from carcasses from different flocks immediately after the final washing step and before the carcasses entered the chilling room (spray chilling). All neck skin samples were taken aseptically with sterile instruments (forceps and single-use scalpels). Immediately after the sampling procedure, the samples were cooled with icepacks and kept refrigerated at around 4°C during transport to the laboratory of the Institute of Food Safety and Food Hygiene of Freie Universität Berlin, Germany. Due to internal work organization processes, all samples were frozen at -30°C for eight weeks before analysis as described in Langkabel et al. [39]. The microbiological analyses were performed according to ISO 4833–2:2013 (+ Cor. 1:2014 + Amd 1:2022) for aerobic plate count at 30°C [40] and to ISO 21528–2:2017 (Corrected version 2018-06-01) for *Enterobacteriaceae* [41] with slight modifications as described in the following. For investigations, neck skin samples underwent a short thawing period of around 30 minutes in a refrigerator at 4°C, so that they were totally thawed. Afterwards, each sample was diluted in Buffered Peptone Water (1:10) and dilution series in sodium chloride peptone agar up to 10^−6^ for aerobic plate count (APC) and to 10^−4^ for *Enterobacteriaceae* counts (EBC) were created. From each dilution, 0.05 ml were plated on the agar surface by dropping on agar plates in duplicate. For APC, Plate Count Agar and for EBC, Violet Red Bile Dextrose Agar was used. Plates were incubated for 48 h at 30°C, then visible colonies were counted as colony forming units (cfu) per gram and the numbers were transformed to logarithmic values to the power of 10 to achieve a normal distribution. The detection limit was set to 2.3 log cfu/g.

Results for APC and EBC are displayed as boxplots. The line within the boxplot shows the median, the box indicates the limits wherein the central 50% of the results fall, whereas the whiskers of each box show the 1.5 times standard deviation limits. Small circles indicate outliers.

### Statistical analyses

Statistical analyses were carried out using IBM SPSS version 24 (SPSS for Windows, IBM^®^—Armonk (New York, USA)). Since the values of APC (cfu/g) turned out to be not normally distributed (visual inspection and Shapiro-Wilk test), the data were transformed to logarithms of base 10. Log cfu/g of APC of LD, LB, the laying hen, and broiler samples were compared using analysis of variance (ANOVA, post hoc Gabriel’s test). Model diagnostics included visual inspection of normality and homoscedasticity of residuals. As the values for log cfu/g of EC were not normally distributed, the Kruskal-Wallis test was performed with Bonferroni correction post hoc test for pairwise comparisons between all groups.

The null hypothesis (H_0_) was that no difference in the microbial load existed between the groups. Significance level was set to 0.05.

### Sensory testing

In November 2017, frozen male and female LD and LB carcasses from the joint research project, as well as frozen broiler carcasses from a conventional broiler abattoir as a control group were sent to the annual testing series at the German Agricultural Society (DLG–Deutsche Landwirtschafts-Gesellschaft) Test Center in Kassel, Germany’s pre-eminent food sensory testing facility. In total, three frozen carcasses from each of the five groups (male LD, female LD, male LB, female LB, and broilers) were included in the sensory testing for fresh poultry meat, following the DLG-5-point-scheme (https://www.dlg.org/en/food/dlg-testcenter-food/what-does-dlg-awarded-mean/testing-methods#c16661). This sensory examination scheme allows objective assessment of impressions recorded with the human senses, and thus, the results obtained are reproducible regarding the quality of the tested products. All products must fulfill special production requirements and were tested in a blinded way, meaning without knowledge of the trademark, name, or other information regarding the producer. The tests were performed by experts specifically trained for sensory tests for fresh poultry meat.

All samples included were prepared following the DLG standard procedure for fresh poultry meat: Whole (defrosted) chicken carcasses were prepared in a roasting tube in a preheated oven at 180°C using the convection mode. For this, each carcass was placed in a plastic roasting tube, two tablespoons of water were added, and the tube was closed. Each tube was pierced three times and heated for 30 minutes. After the core temperature in the roasting tube reached 75°C, the tube was opened carefully so the liquid stayed inside.

All three carcasses from one test group represented one sample in the final testing. The samples were assessed according to the five defined categories (appearance, appearance after roasting, consistency, smell, taste) for the fresh meat test. For each category, individual parameters were checked, and weighting factors were set. Deviations resulted in reduction of points in the 5-point-scheme. Therefore, in the case of no deviations, a parameter was counted as having 5 points and afterwards was multiplied by an individual weighting factor of between one and three, resulting in a total of between 5 and 15 points for each category.

## Results and discussion

### Breed, evisceration results and post-mortem findings

Slaughter was performed in an abattoir for laying hens because pre-tests and results from other research groups from the joint project “Integhof” had shown that male LD chickens did not fit in the machines of a broiler abattoir and were incompletely eviscerated. This was because the LD birds had longer legs than broilers [42] but were smaller overall. Furthermore, LD birds were not as uniform in size and weight as conventional broilers [43–45]. Compared to slaughtering male LD chickens in a conventional broiler abattoir [43], evisceration results for male LD birds in the laying hen abattoir for male LD birds were better, and only a small proportion of carcasses was incompletely eviscerated.

Thus, a laying hen abattoir was used since machines are better equipped to adjust for different types of birds than regular broiler slaughter lines. Normally in laying hen abattoirs, old spent hens, cockerels, and old birds from breeding flocks are slaughtered. These different age groups and bird types have very heterogeneous body conformations. Therefore, machines in the slaughter line need to be adjustable to a broader range of bird sizes and conformations than in broiler abattoirs where carcasses are very homogeneous [46]. Even compared to broilers, we previously observed that male LD birds had a heterogeneous appearance, as was also shown in other studies [42–44]. These authors concluded that the weight range of LD birds was broader compared to a flock of broilers at slaughter, as was also seen in our current study. LD is a new crossbreed and not produced by selective breeding [47–49]. Hence, it is possible that more heterogeneous groups, showing characteristics of the breeds that were crossed, will appear in the first years of breeding; this can be explained by Mendel’s Laws and was shown at the beginning of the 20^th^ century by Punnett and Bailey [50] for the weight of poultry. Assuming that LD would be selectively bred for a longer period, these problems could be minimized, and a more homogeneous group could be fattened. Nevertheless, longer length of the legs and smaller breast muscles compared to broilers [44] might remain as characteristics of the LD breed, and so slaughter of LD chickens should be performed in abattoirs with widely adjustable machinery like that in laying hen abattoirs.

For all three trials in total, official post-mortem inspection resulted in 0.14% (9/6,589) of LB and 0.40% (21/5,344) of LD birds being declared unfit for human consumption. The reasons for unfitness for human consumption of the male LB birds were the occurrence of breast blisters (n = 4), smallness (n = 3), and insufficient bleeding (n = 2). However, the reason for the unfitness for human consumption of the male LD birds in our study was exclusively the small size of the carcasses. This post-mortem finding is not included in the official list of the national meat inspection statistics in Germany where only the findings “inefficient bleeding” and “cachexia” are officially recorded for poultry and hens (see Genesis data bank of DESTATIS: https://www-genesis.destatis.de/genesis/online#astructure). Both findings can be associated with smallness, as too small an animal might not be or might be insufficiently reached by the automatic knife, resulting in inefficient bleeding, while a cachectic carcass might be evaluated as too small. However, as we did not perform the meat inspection ourselves, we cannot ensure such a correlation. Nonetheless, we feel it reasonable to assume that these post-mortem findings of small size that led to condemnation of the carcasses were a specific characteristic of the LD birds studied.

### Microbiological examination

The hypothesis of this study was that microbial loads should be comparable on male LD and male LB poultry as well as on conventionally slaughtered commercial laying hens and broilers.

**Fig 1:**
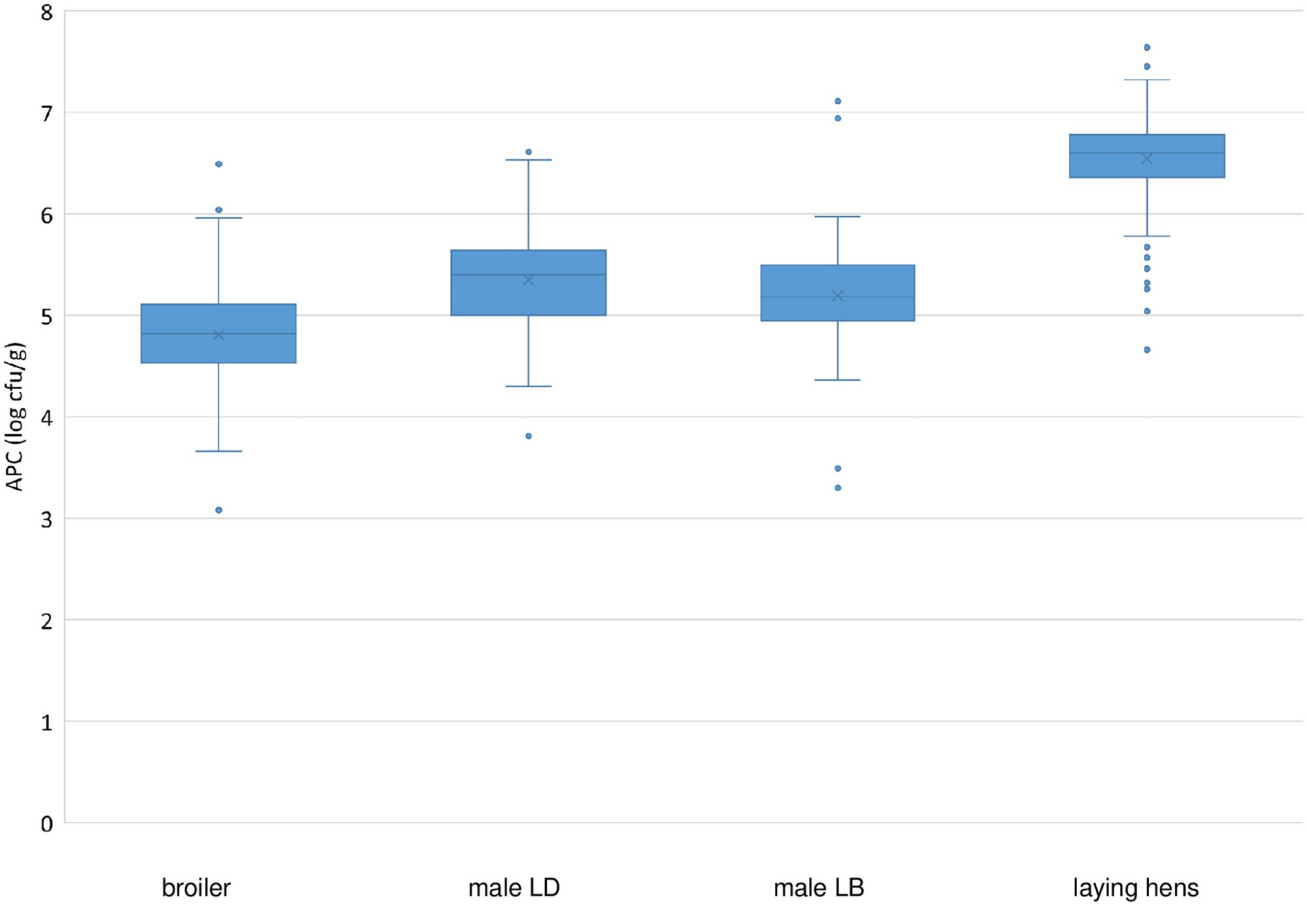
Boxplot of aerobic plate count (APC) (log cfu/g) for neck skin samples of broilers, male Lohmann Dual (LD), male Lohmann Brown Plus (LB), and laying hens.

Fig 1 shows the range and the median values for APC of the sampled carcasses for male LD, male LB, and the control groups consisting of conventional laying hens and broilers.

The mean APC on neck skin before chilling was 5.3 log cfu/g for male LD and 5.2 log cfu/g for male LB birds, while it was 6.5 log cfu/g for laying hens and 4.8 log cfu/g for broilers. These results are in accordance with other studies using neck skin or pooled neck skin and breast skin samples after washing but before chilling, which found mean APC values for broilers ranging from 4.3 log cfu/g to 5.4 log cfu/g depending on the study [51–53] (Table 1). The APC on neck skin of male LD birds slaughtered in a laying hen abattoir are comparable to APC loads of these other types of samples from broilers.

**Table 1:** Mean aerobic plate count (APC) and *Enterobacteriaceae* count (EBC) (log cfu/g) on neck skin samples collected after washing at the end of the slaughter line in our study compared to literature findings.

Source	sample type	APC (log cfu/g)	EBC (log cfu/g)
male LD (present study)	neck skin	5.30	3.30
male LB (present study)	neck skin	5.20	3.30
laying hens (present study)	neck skin	6.50	3.80
broiler (present study)	neck skin	4.80	3.10
[51]	neck skin	5.40	4.60
[52]	neck skin	Plant 1: 5.10	Plant 1: 3.77
		Plant 2: 4.95	Plant 2: 3.67
[53]	pooled sample of neck skin and breast skin	Abattoir A: 4.30	Abattoir A: 3.25
		Abattoir B: 4.38	Abattoir B: 3.35
		Abattoir C: 4.30	Abattoir C: 2.93
[54]	pooled sample of neck skin and breast skin	-	3.34

LD–Lohman Dual (dual-purpose chicken line)

LB–Lohmann Brown Plus (conventional laying line)

In a first ANOVA, the APC results for male LD, male LB, and the control groups (laying hens and broilers) were compared. The ANOVA with post hoc pairwise comparisons showed a global p-value of p < 0.001, meaning that statistically significant differences existed between the pairs. The post hoc Gabriel’s test showed statistically significant differences between the laying hens and the male LD and the male LB and the broilers (p < 0.001), with higher microbial loads for laying hens. Differences between male LD and male LB were not statistically significant (p = 0.251). Differences between male LD and broilers, as well as between male LB and broilers were statistically significant (p < 0.001) (Table 2), but the mean difference was only 0.5 log cfu/g at maximum. Following Hübner et al. [55] (cited in [56, 57]), in principle, an uncertainty of ± 0.5 log levels for microbiological methods can be assumed. So, we conclude that the APC on neck skin of male LD slaughtered in a laying hen abattoir can be seen as comparable to the APC on broiler neck skin. Here, we note that broilers are the typical source of fresh poultry meat and products.

**Table 2:** Comparison of mean aerobic plate count (APC) and *Enterobacteriaceae* count (EBC) in broilers, male Lohmann Dual (LD), male Lohmann Brown Plus (LB), and laying hens.

groups compared	APC	EBC
broiler–LD	p < 0.001	p = 0.070
broiler–LB	p < 0.001	p > 0.999
broiler–laying hens	p < 0.001	p < 0.001
LD–LB	p = 0.251	p > 0.999
LD–laying hens	p < 0.001	p < 0.001
LB–laying hens	p < 0.001	p < 0.001

p < 0.005—significant

LD–Lohman Dual (dual-purpose chicken line)

LB–Lohmann Brown Plus (laying line)

**Fig 2:**
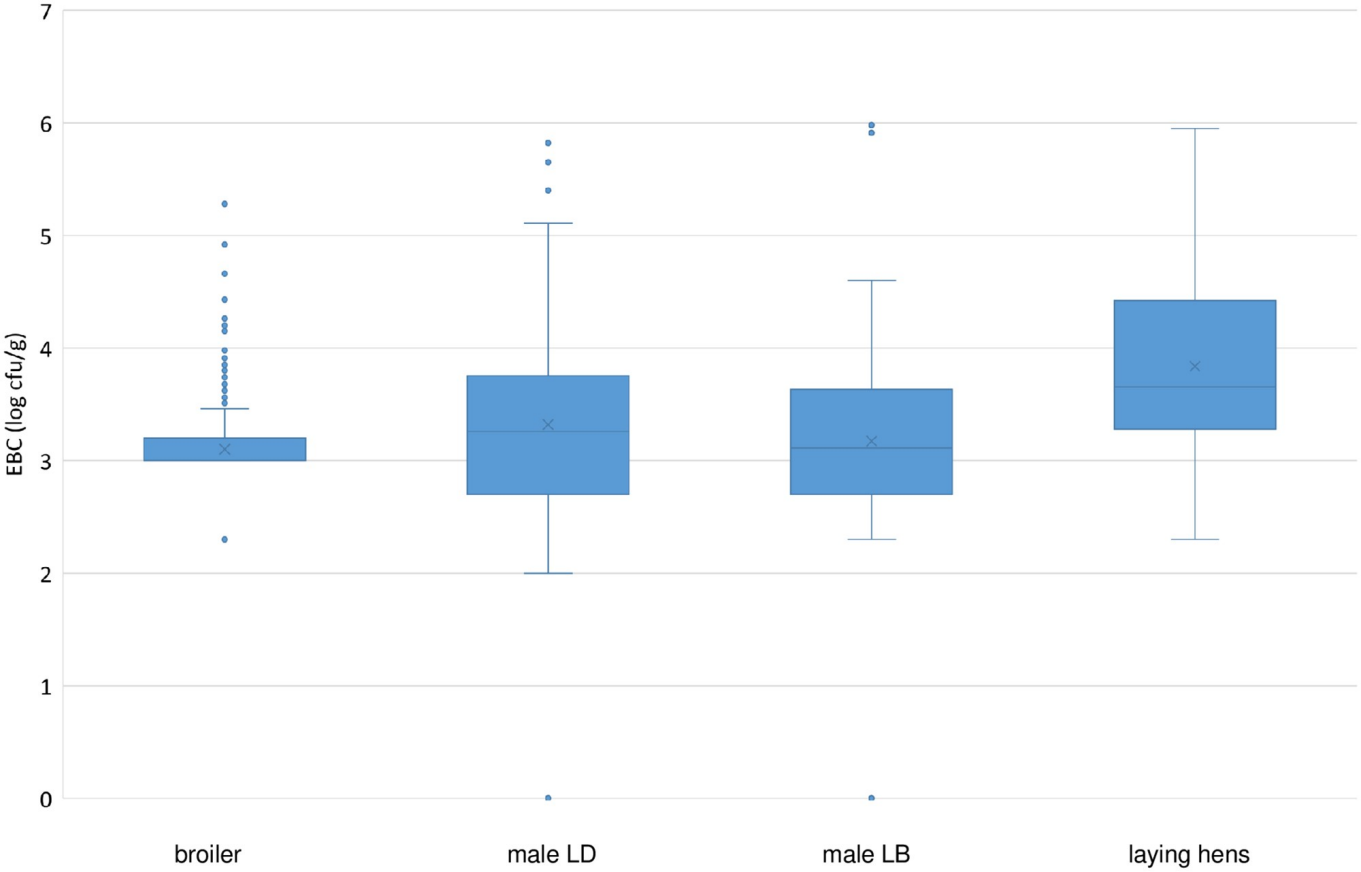
Boxplot for *Enterobacteriaceae* counts (EBC) (log cfu/g) for neck skin samples of broilers, male Lohmann Dual (LD), male Lohmann Brown Plus (LB), and laying hens.

Mean EBC values were 3.3 log cfu/g for both male LD and male LB birds, 3.8 log cfu/g for laying hens, and 3.1 log cfu/g for broilers (Fig 2).

Again, laying hens had the highest microbial loads, and our results for male LD birds were similar to those of other studies analyzing neck skin (pooled neck skin or combined neck and breast skin samples after washing), wherein mean EBC ranged from 2.93 log cfu/g to 4.60 log cfu/g depending on the study [51–54] (Table 1).

The Kruskal-Wallis test was performed for EBC, and the global p-value of p < 0.001 pointed to significant differences existing between the pairs. Bonferroni correction post hoc test showed statistically significant differences for laying hens compared to the other groups (male LD, male LB, and broilers) (p < 0.001), meaning the mean EBC level on neck skin of laying hens was higher than in the other groups. However, there were no statistically significant differences between EBC of male LD, male LB, and broilers (all groups compared with each other) (Table 2).

Our results for male LD birds slaughtered in a laying hen abattoir were within the ranges of reported process hygiene parameters at broiler abattoirs before chilling [51–54]. Therefore, we conclude that the hygiene of the male LD carcasses can be presumed comparable with the hygiene of conventional broilers (regarding APC and EBC status).

### Sensory tests

**Fig 3:**
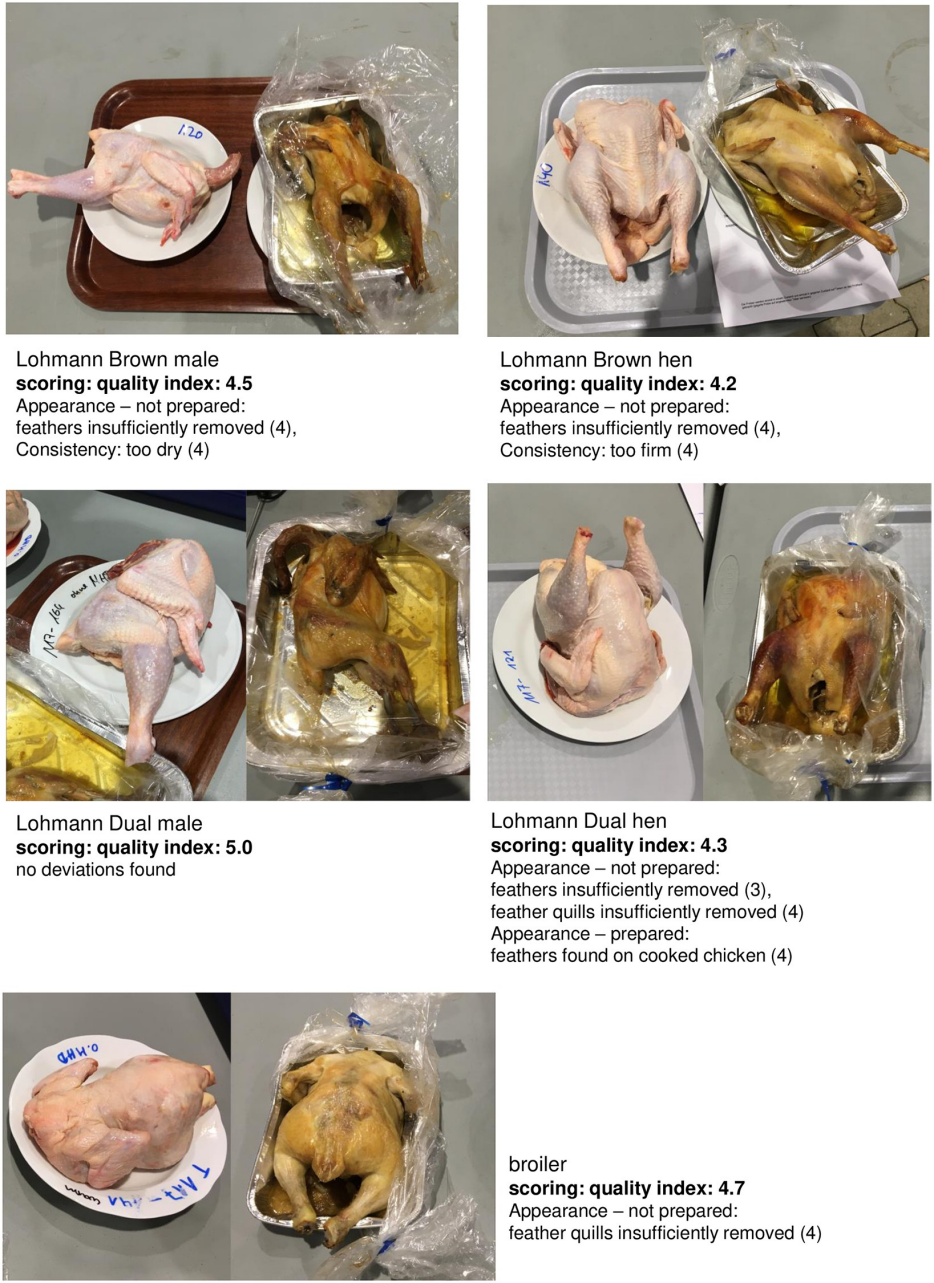
DLG test results.

All carcass samples of all five types of poultry lines examined (male LD, female LD, male LB, female LB, and broilers) produced good to very good results in the sensory quality assessment (Fig 3). The main deviations found resulted in slight point reductions in the category “appearance not prepared”, mainly because of problems with defeathering, and in the category “consistency” for LB birds (males and females). For all other categories, no deviations were found, which resulted in maximum points for each of these categories for each test group. Showing the fewest sensory abnormalities, male LD were ranked highest (i.e., best), ahead of broilers, female LD, and male and female LB.

In the joint project “Integhof”, another research group also examined sensory aspects of meat from male LD compared to Ross broiler meat [37]. Their panel group consisted of ten members experienced in sensory description of pork and poultry products. In contrast to DLG testing, Siekmann et al. [37] found that muscle samples of LD birds were significantly more tender and juicier than (Ross) broiler muscle samples.

Our exemplarily performed tests at DLG yielded good sensory results for male LD compared to the other groups tested and showed this meat would be comparable with conventional broiler meat on the market. However, since only a small number of samples were subjected to sensory testing, results cannot be generalized, and further studies should be conducted to investigate the influences of genetics, feed, and age.

## Conclusions

1. Slaughter of the male dual-purpose birds in a laying hen abattoir was successful compared to previous data demonstrating problems with slaughter of dual-purpose birds in broiler slaughter lines.
2. Even though the male LD birds showed more variation in body composition, APC and EBC results for male LD birds slaughtered in a laying hen abattoir were within the range of comparable reported process hygiene results at broiler abattoirs before chilling. Therefore, the hygiene status of the male LD birds before chilling is comparable to that of conventional broilers.
3. In terms of ethical acceptability, microbial status, and sensory characteristics, as an alternative to broilers, LD as a new dual-purpose line can be considered when the specific requirements for their slaughter are taken into consideration.

## Supporting information

S1 File (XLSX)
